# Supplementary figures and images for: Heterologous expression of a Glyoxalase I gene from sugarcane confers tolerance to several environmental stresses in bacteria
Source: PeerJ. 2018 Oct 31;6:e5873. doi: 10.7717/peerj.5873 (PMC6215438; doi:10.7717/peerj.5873)

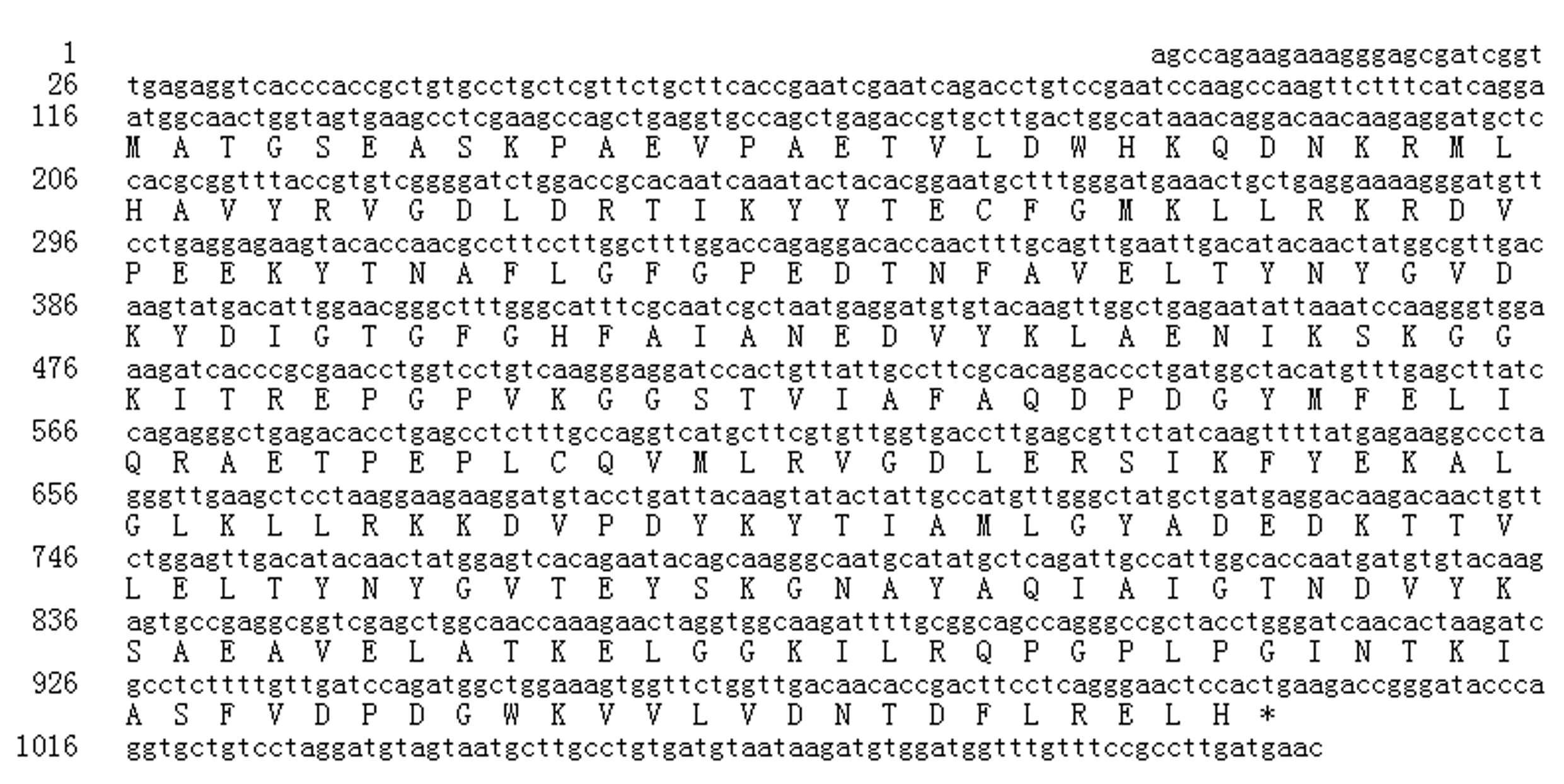

Supplement: Figure S1 [file peerj-06-5873-s001.png]

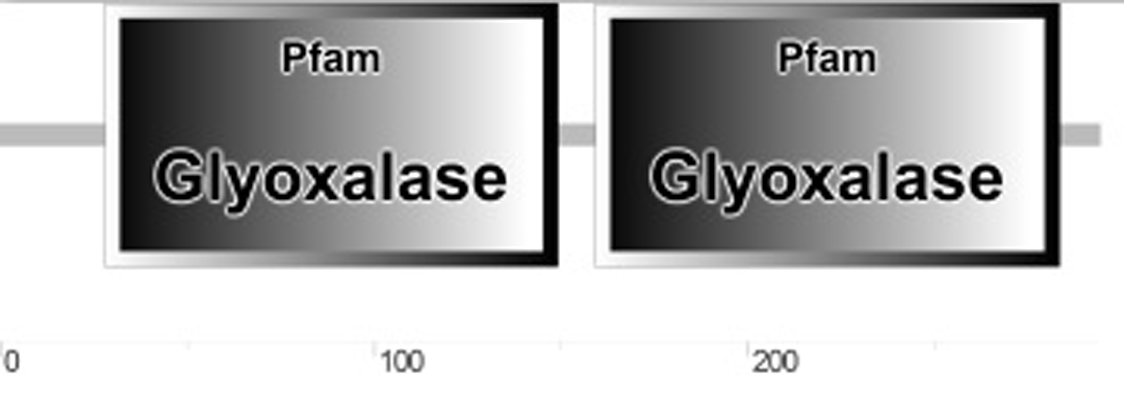

Supplement: Figure S2 [file peerj-06-5873-s002.png]

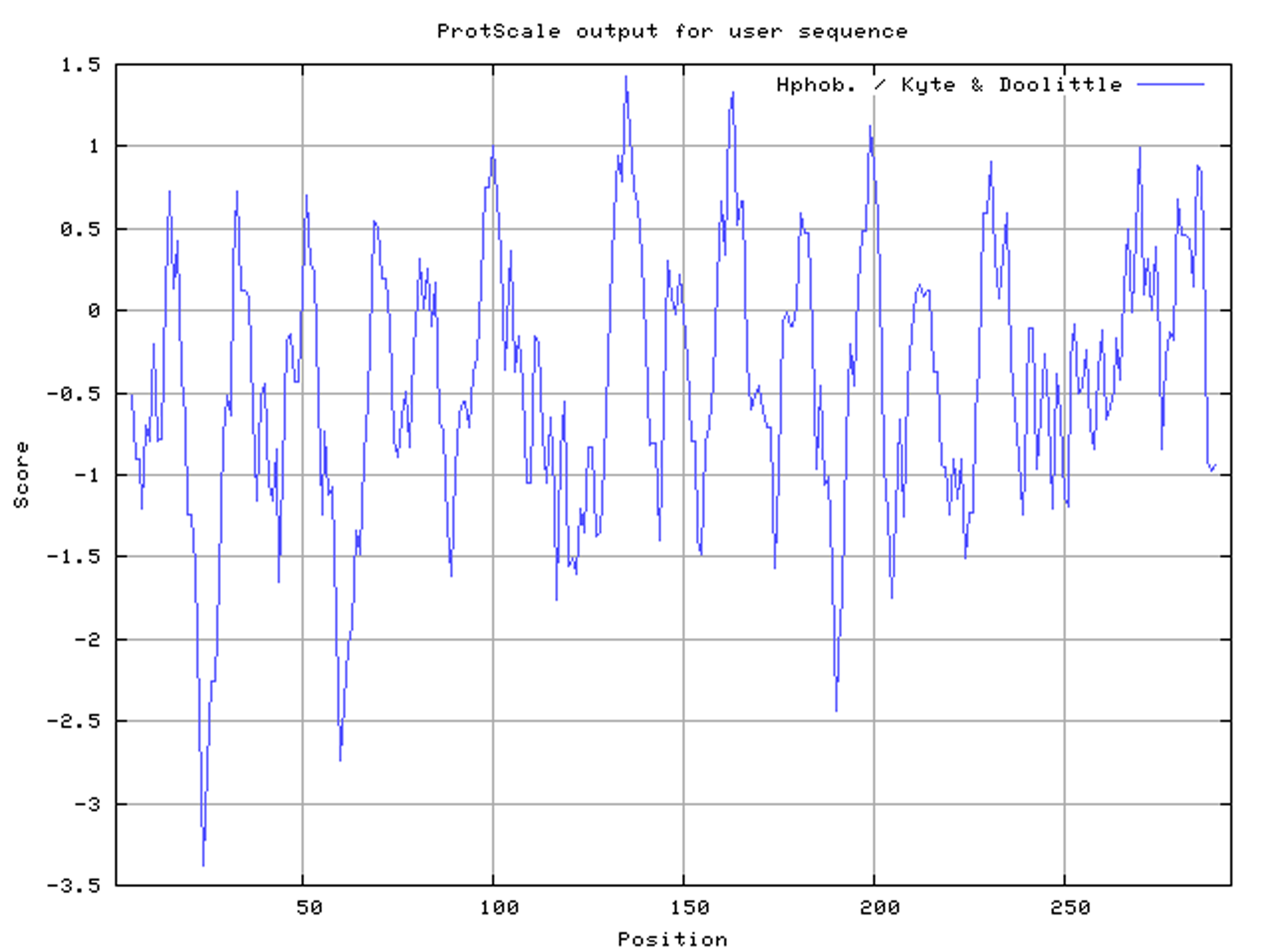

Supplement: Figure S3 [file peerj-06-5873-s003.png]

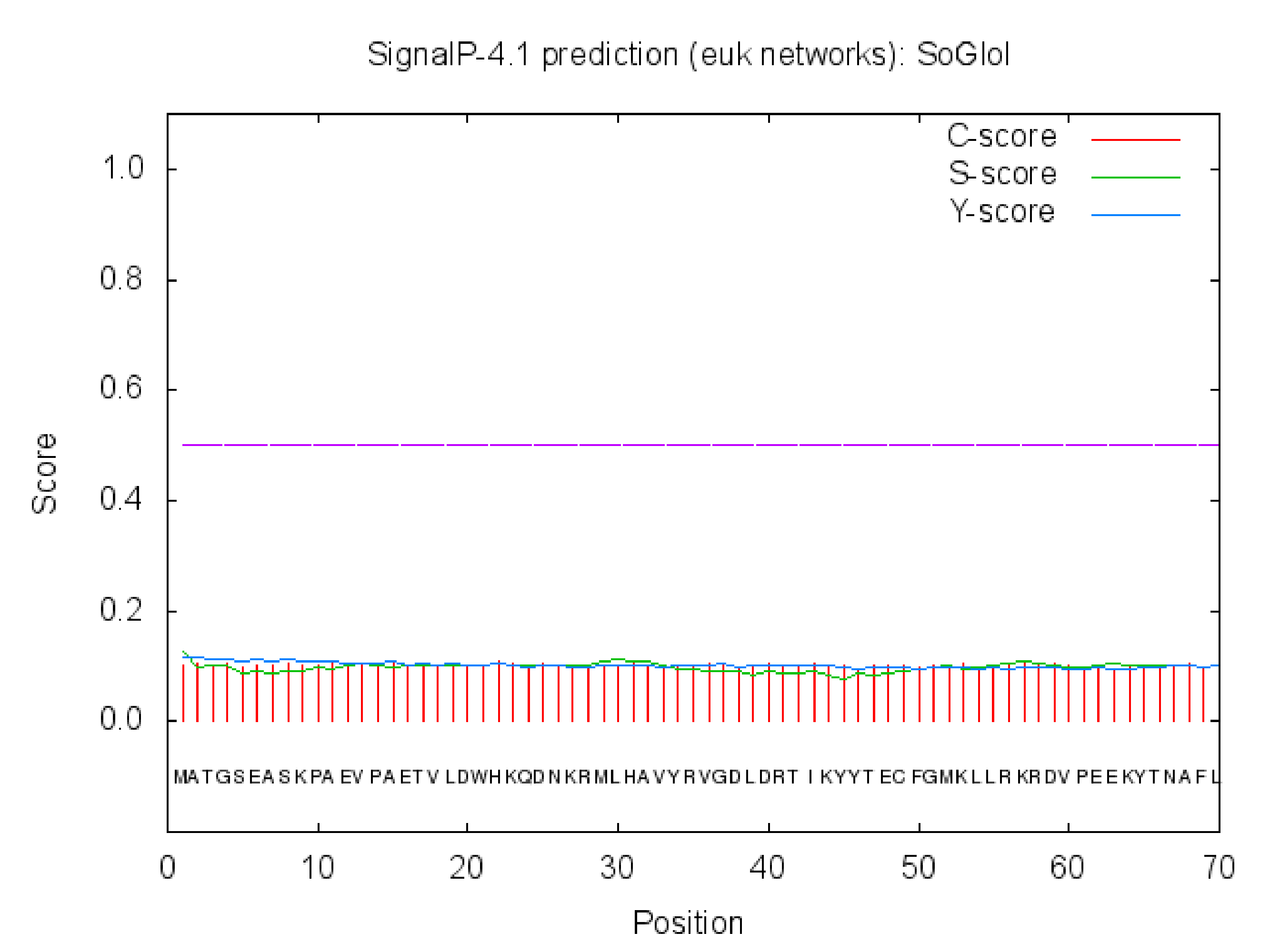

Supplement: Figure S4 [file peerj-06-5873-s004.png]

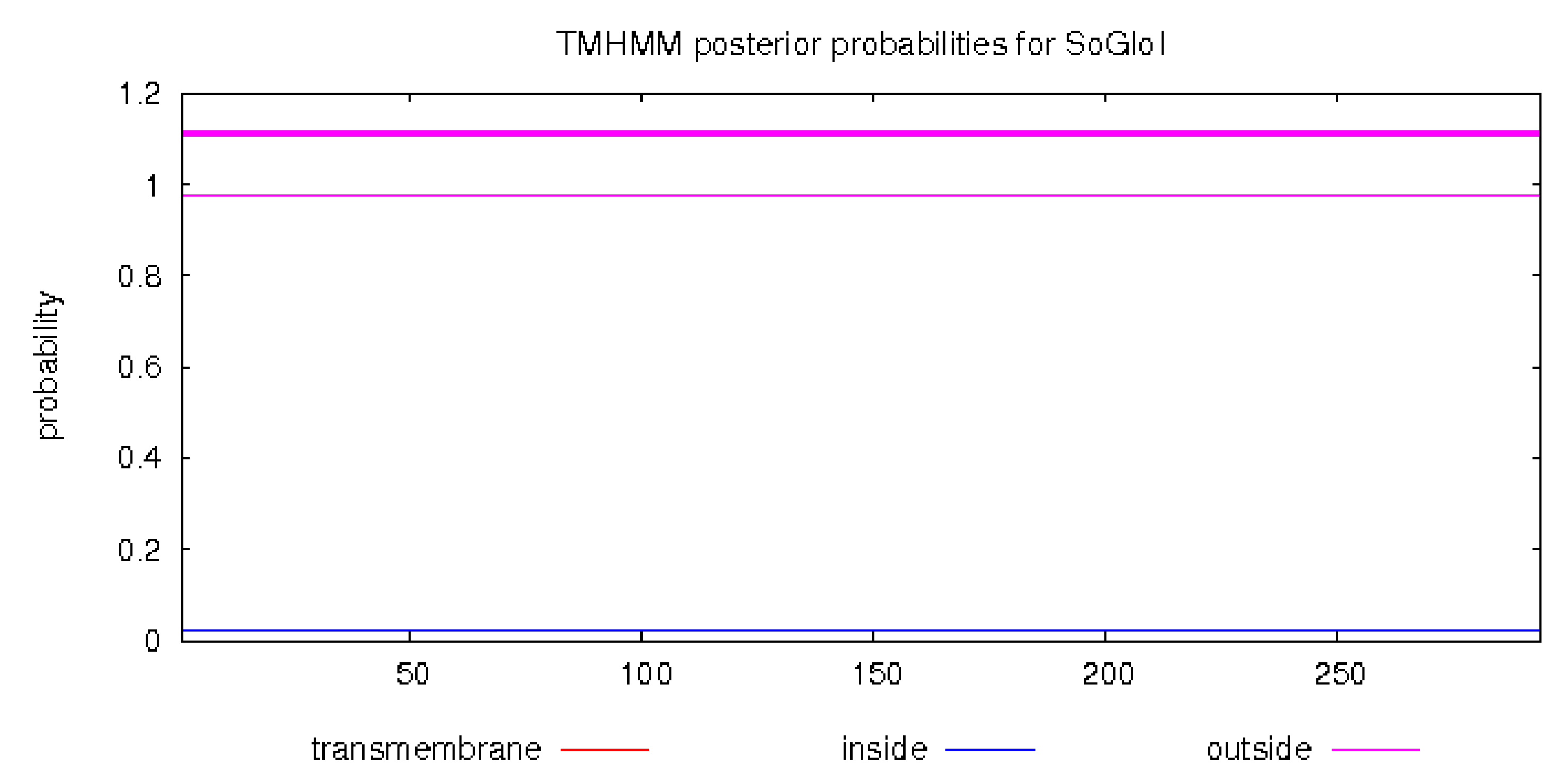

Supplement: Figure S5 [file peerj-06-5873-s005.png]
